# Recapturing embryonic potential in the adult epicardium: Prospects for cardiac repair

**DOI:** 10.1002/sctm.20-0352

**Published:** 2020-11-21

**Authors:** Andia N. Redpath, Nicola Smart

**Affiliations:** ^1^ Department of Physiology, Anatomy and Genetics, British Heart Foundation Centre of Regenerative Medicine, Burdon Sanderson Cardiac Science Centre University of Oxford Oxford UK

**Keywords:** cardiac, cell biology, developmental biology, differentiation, progenitor cells, tissue regeneration

## Abstract

Research into potential targets for cardiac repair encompasses recognition of tissue‐resident cells with intrinsic regenerative properties. The adult vertebrate heart is covered by mesothelium, named the epicardium, which becomes active in response to injury and contributes to repair, albeit suboptimally. Motivation to manipulate the epicardium for treatment of myocardial infarction is deeply rooted in its central role in cardiac formation and vasculogenesis during development. Moreover, the epicardium is vital to cardiac muscle regeneration in lower vertebrate and neonatal mammalian‐injured hearts. In this review, we discuss our current understanding of the biology of the mammalian epicardium in development and injury. Considering present challenges in the field, we further contemplate prospects for reinstating full embryonic potential in the adult epicardium to facilitate cardiac regeneration.

## Graphical abstract

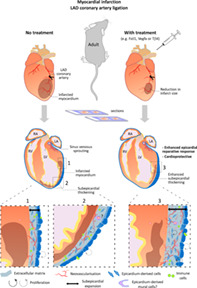

**Significance statement**

Determining how to optimally manipulate the adult epicardium for cardiac repair requires a better understanding of its intrinsic properties, both in development and injury. This article outlines processes documented to occur in the adult epicardium postinjury and reveals gaps in knowledge of the mammalian tissue. Despite great strides made in the past two decades, the secretome and crosstalk between the epicardium and its neighboring cells, including those in transit through the tissue, are largely unexplored, yet important. The field is also troubled by controversies surrounding innate epicardial fate postinjury (and in development), and the authors deliberate how current tools necessitate compromise until alternatives are identified. This perspective summarizes recent literature that utilizes new technologies bound to hasten the quest to harness the epicardium for repair.

## 1 INTRODUCTION

The recognition of the regenerative capacity of lower vertebrate^1^ and neonatal mammalian^2^ hearts has reinvigorated the search for endogenous reparative pathways that may be translated to regenerate the human heart after injury. Such pathways emulate the complex, coordinated events that occur during embryonic development, upon which our understanding of cardiac formation and composition is founded.^3^ The adult mammalian heart lacks regenerative ability, owing to the absence of tractable cardiomyocyte (CM) precursors and the inability of mature CMs to proliferate after injury.^4^, ^5^, ^6^

Heart failure is commonly caused by myocardial infarction (MI), with major morbidity and mortality consequences worldwide.^7^ Following MI, damaged CMs are replaced by an expansion of tissue‐resident cardiac fibroblasts (CFb), which respond by transitioning to myofibroblasts, depositing collagen to the fibrotic scar^8^, ^9^, ^10^ to prevent ventricular wall rupture.^9^, ^11^ However, excessive fibrosis impairs contractile function and initiates a deleterious cycle of myocardial loss and adverse remodeling.^12^ Strategies to limit myocardial damage (eg, restoring adequate perfusion,^13^ replacing dying CMs^4^, ^5^ and reducing unwarranted fibrosis^14^, ^15^) are thus vital to prevent heart failure.

A central regulator of the processes outlined above, and therefore an important therapeutic target, is the epicardium. Commonly described as the outermost layer of the heart in vertebrates, this mesothelial tissue is a source of multipotent progenitors, growth factors, and extracellular matrix (ECM) components during cardiac development and following injury.^16^ In this review, we discuss the prospects for reinstating embryonic potential in the adult epicardium to facilitate cardiac regeneration. We will highlight recent literature and new technologies that are proving invaluable in the quest to harness the epicardium for repair.

## 2 RECAPTURING EMBRYONIC POTENTIAL

### 2.1 Epicardium: Developmental origin, formation, and function

The epicardium originates from a transient structure in the developing embryo called the proepicardial organ (PEO), adjacent to the septum transversum, and proximal to the looping heart tube and sinus venosus (SV).^3^, ^17^, ^18^ Lineage tracing studies in the mouse suggest that the PEO derives from *Nkx2.5* and *Isl1* expressing lateral plate mesoderm, the source of most other cardiac precursor cells.^19^ PEO formation is induced by a carefully controlled balance of bone morphogenetic proteins (BMPs) and fibroblast growth factors (FGFs), which determine whether LPM adopts a myocardial or epicardial fate.^17^ From embryonic day (E)9.5 in mouse, epicardial progenitors residing in the PEO detach and migrate to envelop the developing myocardium. Epicardial formation from the PEO is widely conserved, having been described in all vertebrate species examined, including zebrafish,^20^ Xenopus,^21^ chicken,^22^ mice,^23^ rats,^24^ and humans.^25^ Due to the scarcity of available tissue to study early human embryology, insights into the formation of the human epicardium are limited. However, examination of carnegie stage (CS) 12 embryonic sections by light microscopy first revealed villous protrusions of mesothelial cells extending from the sinus wall onto the dorsal side of the ventricle and spreading over the heart as a squamous epithelium,^25^ supporting a conserved mechanism. Histological analysis of CS11 embryos (4 weeks postcoitum, equivalent to mouse E10) suggested that epicardial formation was already underway at this earlier stage, with “round, progenitor‐like cells” described to overlie the compact myocardial layer, albeit these cells were not distinguished by marker analysis and the PEO protrusions were not captured in these samples.^26^ Epicardial formation is complete in human embryos by CS15 and is characterized by expression of markers, such as WT1, TCF21, GATA5, TBX18, cytokeratin, and podoplanin,^26^ consistent with other species. Unlike the monolayer structure in rodent, chick, and zebrafish hearts, the multilayered human epicardium consists of a mesothelium overlying connective tissue and an expanded subepicardial space containing elastic fibers and blood vessels.^27^ This species difference emerges during fetal stages^26^, ^27^ and, during adulthood, adipose tissue depots accumulate within the subepicardial space, which can profoundly influence cardiac function.^28^

The extent and depth of our understanding of epicardial origin, formation, and role in supporting mammalian cardiac development is due, in large part, to the ease of genetic manipulation of the mouse embryo, for lineage tracing and “knockout” developmental studies. The epicardium serves three primary functions to support cardiac development.

#### 2.1.1 Direct cellular contribution

**FIGURE 1:**
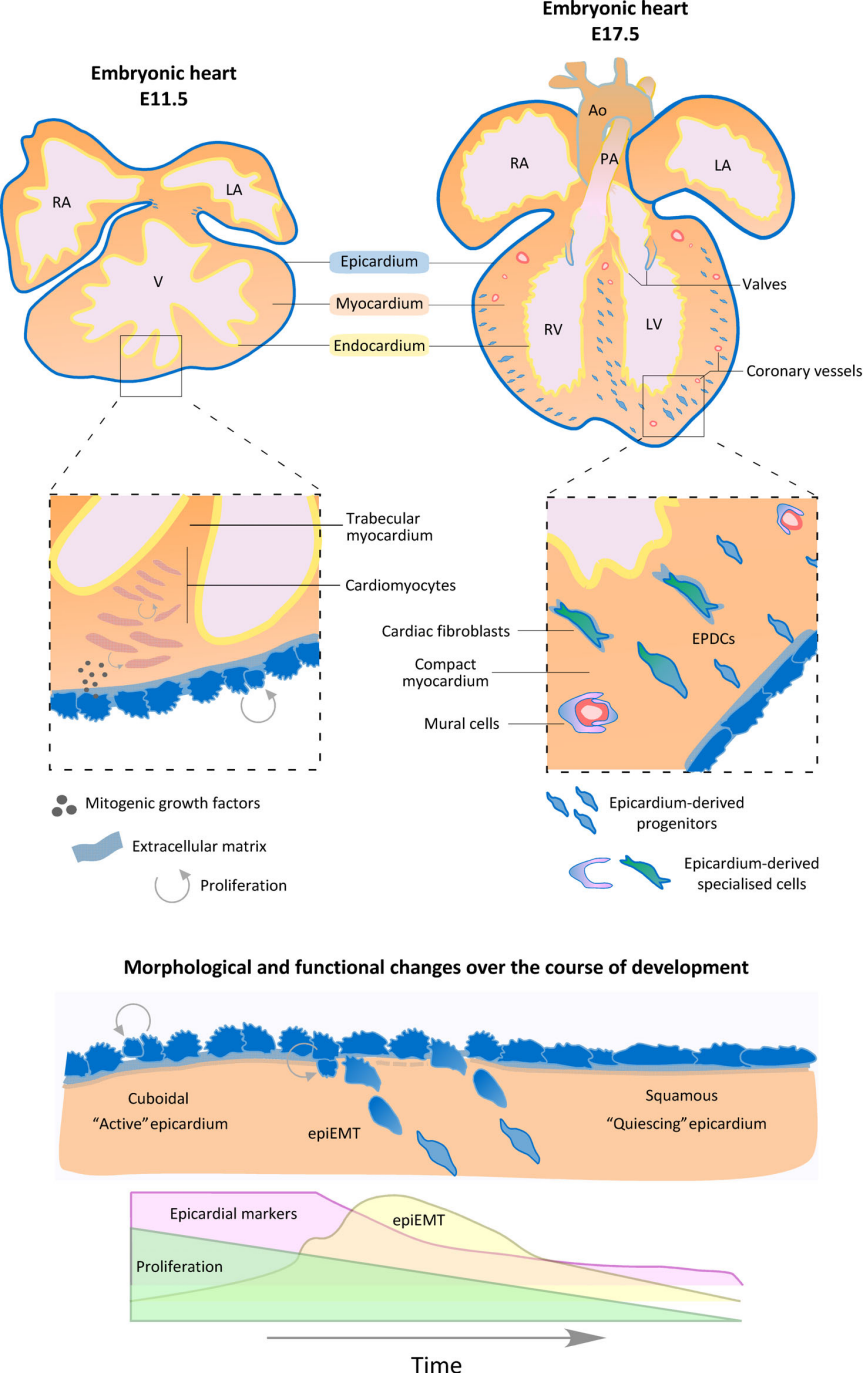
Epicardium formation and function. The epicardium forms the outermost layer of the embryonic heart, and almost completely envelops the myocardium from embryonic day (E)11.5 in mouse. The embryonic epicardium is characteristically “active” with high proliferation, and elevated generation of mitogenic growth factors and extracellular matrix components which support cardiomyocyte propagation and maturation. As development progresses, epicardial cells undergo epithelial‐mesenchymal transition (epiEMT) to provide epicardium‐derived progenitors, precursors for epicardium‐derived specialized cells such as cardiac fibroblasts and mural cells. Epicardial derivatives—transitory and differentiated progeny—are grouped under the term epicardium‐derived cells (EPDCs). By E17.5, the “quiescing” epicardium forms a continuous layer of cells with squamous morphology, diminished proliferation, epicardial marker expression and epiEMT. Ao, aorta; LA, left atrium; LV, left ventricle; PA, pulmonary artery; RA, right atrium; RV, right ventricle; V, ventricle

Prior to maturation of the epicardial layer, successive, regionalized waves of epithelial‐mesenchymal transition (EMT) between E11.5 and E13.5 mobilize epicardium‐derived mesenchymal cells, as precursors for atrioventricular valve mesenchyme, CFb and mural cells (pericytes and vascular smooth muscle cells [vSMCs]).^16^, ^29^ A variety of myocardial‐derived signals promote EMT, including transforming growth factor‐β (TGFβ) and FGFs, although roles specifically in EMT have been difficult to distinguish from roles in migration and fate, as the processes are intricately linked.^30^ Progenitors and specialized cells originating from the epicardium are commonly referred to as epicardium‐derived cells (EPDCs; Figure 1). Early lineage tracing studies supported further contributions extending to CMs^31^, ^32^ and coronary endothelial cells (CECs).^32^, ^33^ While constitutive and inducible genetic lineage tracing models in mouse have, in many ways, helped advance our knowledge of the epicardium, they have also contributed toward a muddled narrative and dispute surrounding the extent to which certain fates are adopted. Genetic lineage tracing is predicated on the major assumption that the genetic marker used to drive labeling of the parent progenitor cell is specific and neither independently expressed in its differentiated progeny (derivatives) at later stages, nor in neighboring cell types.^3^ Collectively, research over the last two decades has revealed that epicardial markers, taken individually and particularly in the absence of efficient temporal labeling methods, do not meet these criteria. Namely, *Tbx18* and *Tcf21* are expressed both in epicardial derivatives and in nonepicardially contributed cell types,^18^, ^34^, ^35^, ^36^ whereas *Wt1* and *Sema3d* are expressed in nonepicardial cell types that populate the heart later in development, as summarized in Table 1.^18^, ^37^, ^38^ A reappraisal of marker specificity and the utility of fate mapping tools has resulted in a consensus that the epicardium is an unlikely native source for CMs and CECs.^6^, ^18^ Future studies would benefit from utilizing improved models based on (a) dual lineage tracing^39^ and (b) specific epicardial markers (e.g., Upk3b).^18^, ^40^, ^41^, ^42^

**TABLE 1:** Example studies that demonstrate nonepicardial expression domains of common epicardial markers in the developing heart

Marker expression domains	
Epicardial derivatives	Other cell types (nonepicardial origin)
*Tbx18*—vascular smooth muscle cells (vSMCs)^18^, ^36^ *Tcf21*—mesenchymal cells and cardiac fibroblasts (CFbs)^18^, ^34^	*Tbx18*—vSMCs (neural crest derived, base of heart)^18^, ^36^ and cardiomyocytes (CMs)^18^, ^35^ *Tcf21*—CFbs^18^ *Wt1*—coronary endothelial cells (CECs)^18^, ^37^, ^38^ *Sema3d*—lymphatic endothelial cells^18^

#### 2.1.2 A source of essential paracrine signals

The secretory repertoire of the embryonic epicardium has been elaborated over the years to include an extensive list of growth factors, morphogens, and chemokines that mediate CM proliferation (e.g., IGF2^43^ and BMP4^41^) and coronary vessel growth (e.g., Elabela^44^ and CXCL12^45^). The embryonic epicardial secretome has been reviewed at length elsewhere^46^; however, a more comprehensive list will soon emerge, with recent advances in “omics” and single‐cell resolution technology^47^ leading to a surge of data profiling the epicardial transcriptome. Li et al^41^ identified *Rspo1* expression in the epicardium—an activator of the canonical Wnt signaling pathway—from single‐cell RNA sequencing (scRNA‐seq) analysis of E10.5 hearts. The authors applied ligand‐receptor analysis to identify hundreds of paracrine signals through which the epicardium communicates with CMs, endothelial/endocardial, and mesenchymal cells. R‐spondin‐1 and BMP4, ligands mined from this data set, were proposed to jointly stimulate proliferation of the compact myocardium.^41^ It is important to note that further work will be required to experimentally validate putative cell‐cell interactions, especially to delineate regionalized, protein‐level crosstalk mechanisms.

#### 2.1.3 Dynamic regulation of the cardiac ECM

In addition to providing physical support for tissues, ECM molecules participate in cell‐cell communication, by acting as a reservoir for ligands and essential co‐receptors for signaling pathways. Thus, ECM confers the strict spatiotemporal regulation required for cardiac morphogenesis^48^ and the cardiac mesenchyme is a central hub for ECM components. While epicardial contribution to ECM remodeling via its derivative mesenchymal and CFb progeny is widely accepted, the direct role of epicardial cells in ECM deposition and turnover is understudied, yet clearly important. A cell‐autonomous role in formation and modification of the surrounding ECM landscape, including fibronectin fibrils, drives autocrine regulation of epicardial EMT (epiEMT) and, moreover, is postulated to signal myocardial growth and compaction, and to provide a foundation for coronary sprouting from the SV.^49^ The importance of these features will be described in the next section.

### 2.2 Epicardium: Native friend or foe in cardiac injury?

**FIGURE 2:**
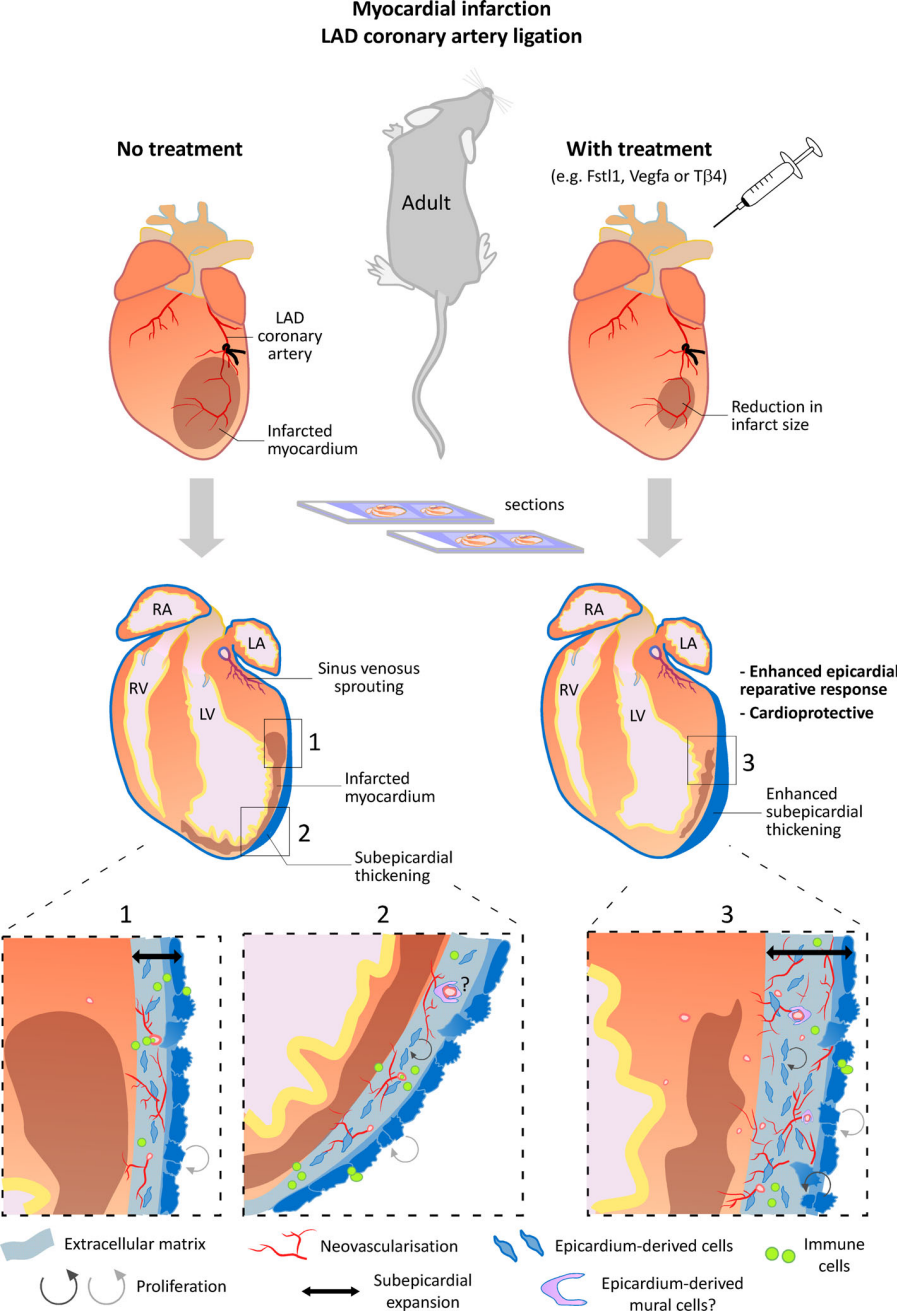
Treatments identified to enhance epicardial activity are cardioprotective. A routine model of myocardial infarction (MI) is achieved through surgical ligation of the left anterior descending (LAD) coronary artery in the adult mouse. Impeding blood supply below the ligated coronary artery creates an infarcted myocardium characterized by cardiomyocyte cell death, fibrosis, and thinning and dilation of the left ventricular (LV) wall. In response to myocardial damage, the epicardium becomes active, proliferative and provides derivatives to form a thickened subepicardium. The extracellular matrix and growth factor rich microenvironment of the expanded subepicardium supports neovascularization and immune cell infiltration. Treatments such as exogenous Thymosin β4 target the epicardium, to augment subepicardial thickening and neovascularization. LA, left atrium; LV, left ventricle; RA, right atrium; RV, right ventricle

Prior to birth, the epicardium downregulates many of its genetic markers^18^ and undergoes morphological changes, from cuboidal to squamous morphology.^50^ It is generally accepted that these events, which include a steady reduction in proliferation,^51^ depict the onset of epicardial quiescence (Figure 1). Thus, with loss of markers and concealed appearance, it is almost impossible to distinguish the ventricular epicardium of the healthy adult mouse heart by histological analysis. Notably, atrioventricular sulcus and atrial epicardium were reported to sustain marker expression into adulthood,^52^ the reasons for this remain unknown. Following cardiac injury, commonly *Wt1* and *Aldh1a2*, are detected throughout the outermost layer, once again distinguishing the ventricular epicardium.^40^, ^52^, ^53^ Albeit transient, their expression represents epicardial reactivation and increased proliferation, accompanied by subepicardial thickening that is most pronounced around the injury site (Figure 2). The subepicardial mesenchyme is similarly transient following injury in adult mouse, as in development, and shown to originate from the reactivated epicardium.^53^, ^54^ The precise roles of the reactivated epicardium in the injured heart, and the extent to which these recapitulate developmental mechanisms, are incompletely understood. While clear differences have been identified between embryonic and post‐MI responses, there is evidence that the epicardium is once again called upon for cellular contribution, paracrine signaling and ECM modulation, as discussed below.

Due to cardiac cell contribution in development, the possible recapitulation of these cell fates by the reactivated adult epicardium was largely assumed but only partially investigated. Earlier work demonstrated significant contribution to CMs, CFbs, CECs, and mural cells, suggesting that the adult epicardium preserves its cellular plasticity.^52^, ^53^ However, with revised interpretation and more cautious use of lineage tracing models, both in the neonate and adult, an epicardial origin appears unlikely, with the majority of de novo CFbs, CECs, vSMCs, and rare CMs post‐MI seemingly arising from their respective preexisting resident populations.^6^, ^8^, ^9^, ^11^, ^55^, ^56^, ^57^ Caveats relating to the specificity of embryonic epicardial markers, as discussed previously, equally apply in the adult,^9^, ^36^, ^37^ rendering available *constitutive* epicardial lineage tracing lines unreliable. Few studies demonstrate epicardium‐derived cellular contribution and, without exogenous stimulation, the extent of de novo contribution is minimal.^53^, ^54^, ^58^, ^59^ Zhou et al^53^ first utilized the inducible Wt1Cre^ERT2^ mouse line to trace the epicardium and its progeny post‐MI. Tamoxifen was administered before MI,^53^ to minimize labeling of CECs which upregulate *Wt1* in response to injury.^37^ However, the disadvantage of such an approach is that only a small fraction of resting epicardial cells are labeled, with the Wt1‐expressing, injury‐reactivated population largely unlabeled. While a proportion of labeled derivatives were found to express NG2^54^ and αSMA,^52^, ^53^ ostensibly contributing pericytes and vSMCs, respectively, the subepicardial and border zone vasculature remained largely untraced.^54^ Notwithstanding the inefficient labeling noted above, the scarcity of fate mapped cells implies a predominantly nonepicardial origin for neovessels of the infarcted heart. The epicardium may be a major source of CFbs during development^60^, ^61^ but, when reactivated, seems not to be responsible for de novo CFbs, which contribute to the scar.^8^, ^60^, ^62^ If exogenous treatments can be used to augment epicardium‐derived cell differentiation, as discussed later, a greater understanding of cell fate is necessary to ensure precursors commit to beneficial cell types, rather than enhance the CFb‐myofibroblast pool.

In mice, epicardial activity post‐MI has been implicated in both beneficial^53^, ^54^, ^58^, ^63^, ^64^ and detrimental^40^ effects. Duan et al,^63^ in a model of ischaemia‐reperfusion injury, demonstrated that hindering epicardial activation, subepicardial thickening and CFb pro‐fibrotic response worsened cardiac function.^63^ Other mouse studies have established numerous pro‐reparative neovascular qualities, through recruitment of vascular support cells and the production of angiogenic factors and ECM‐rich microenvironment.^53^, ^54^ Literature supporting these properties—drawing on useful comparisons with the embryonic program—has been reviewed elsewhere.^13^, ^65^ In contrast, Huang et al^40^ suggested a harmful pro‐inflammatory and pro‐fibrotic role for the epicardium, with improvement in cardiac function upon disruption of CCAAT/enhancer‐binding protein (C/EBP)‐mediated activation of epicardial gene enhancers, for example, *Wt1*.^40^ The actin‐binding peptide Thymosin β4 (Tβ4), shown to stimulate epicardial‐mediated regeneration,^58^, ^66^, ^67^ was found to interact with the C/EBP‐recruited SWI/SNF chromatin‐remodeling complex in order to induce epicardial *Wt1* expression,^68^ and thus, contrary to the above, confirms the therapeutic benefits of augmenting C/EBP activity in the epicardium in disease.

An additional role for the epicardium is emerging, in regulating crosstalk between the immune response and the injured heart, the intricacies of which will need to be better understood in order to promote beneficial, rather than detrimental, outcomes. This is borne out by a study demonstrating an important role for epicardial Hippo signaling in suppressing inflammation post‐MI through enhanced recruitment of regulatory T cells.^64^ The contrasting findings, compared with those of Huang et al,^40^, ^64^ highlight the need to profile the specific immune cell populations that transit through the epicardium, along with the chemokines which attract them. Potential candidates include GATA‐6 expressing pericardial cavity macrophages found to limit fibrosis,^69^ after their recruitment and migration through the activated epicardium post‐MI.

In zebrafish, the epicardium's regenerative role is compelling, with many studies expanding the list of signaling pathways and ECM molecules that participate in the reparative response.^16^, ^70^ Marín‐Juez et al^70^ demonstrated intricate cooperation between epicardial Cxcl12 and endocardial Vegfa signaling, in regulating coronary sprouting and myocardial restoration. Within 24 hours, superficial coronary sprouts formed and were guided through the (sub)epicardium via Cxcl12 signaling, stimulated by hypoxia.^70^ However, it is important to note that many of the regenerative pathways documented in the zebrafish model additionally bring on substantial CM replenishment,^16^ an essential regenerative process^1^ that does not occur naturally in the injured mammalian heart. The fact that the mammalian epicardium responds intrinsically to injury, ostensibly as in zebrafish, is encouraging, but may not be sufficient if the full reparative repertoire, including CM proliferation and neovascularization, cannot be simultaneously harnessed. The following section will examine current challenges and gaps in the field, and advocate new approaches that may assist in epicardial target discovery for clinical translation.

### 2.3 Challenges faced and deep curiosities

If current genetic tools have limited specificity in the embryo, how can we be confident in their use for neonatal or adult epicardial studies? Widely used embryonic epicardial markers, *Tbx18*, *Wt1*, and *Tcf21* all exist as inducible Cre lines and are favored over constitutive lines to assess de novo contribution. However, all three markers are expressed in nonepicardial domains postnatally, irrespective of epicardial origin. *Tcf21* marks most CFbs,^9^, ^11^, ^71^ while *Tbx18* marks all perivascular cells^36^ in the adult mouse heart. Fortunately, only a small proportion of adult CECs express *Wt1* at baseline, confined to larger coronaries,^37^, ^53^ in contrast to the vast majority of CECs which express the gene in embryonic and early neonatal hearts.^18^, ^37^, ^38^, ^53^ This makes the inducible *Wt1*CreER line the only credible option currently available for adult studies, with the important caveat that induction should be temporally restricted to avoid significant CEC targeting postinjury,^37^, ^72^ even though the restricted temporal window reduces the efficiency of recombination. The Wt1Cre^ERT2^ line was used in the embryo to efficiently label the epicardium (~89% labeling with induction at E9.5, and just over 3% of CECs labeled^18^). Zhou et al^53^ estimated that the resting adult epicardium comprises 25% WT1+ cells and 50% of these were successfully labeled (just 13% of epicardial cells) in healthy Wt1Cre^ERT2/+;^Rosa26^mTmG/+^ hearts, however, whether this is representative of the whole ventricular epicardium is unclear.^53^ This would suggest that adult epicardium‐derived de novo contribution has not been adequately assessed because of insufficient labeling at baseline. To overcome this limitation, genes expressed specifically and constitutively in the quiescent adult epicardium should be identified to generate new inducible Cre driver lines. Candidate epicardial genes such as those already identified from transcriptomic studies^18^, ^41^, ^73^ may be pursued. Alternatively, the existing Wt1 Cre line may be paired with dual lineage tracing tools to widen the window for tamoxifen induction.^39^ While on the subject of inducible epicardial Cre lines, we feel obligated to mention the general issues surrounding efficiency of recombining floxed genes^74^ for knockout studies, which is particularly problematic during development. We recommend that percentage knockout or knockdown be assessed at the level of target gene expression—as opposed to relying solely on reporter labeling as a readout—to enable accurate interpretation of results. This is especially important when assessing genes that may be involved in epicardial cell survival and fate decision, since there may be potential for nontargeted cells to expand and compensate for the targeted population.

Despite significant findings in terms of molecular regulators,^40^, ^68^ the upstream signals that instruct epicardial activation and other early cellular responses to injury remain uncharacterized in mammals. Van Wijk et al^52^ suggested that the epicardial layer covering the infarct was severely damaged at day 1 post‐MI, demonstrated by the absence of Wt1‐lineage‐labeled cells (constitutive Wt1^Cre^; Rosa26 Reporter line) and compromised tissue integrity.^52^ In contrast, Huang et al^40^ reported strong epicardial enhancer activity overlying the infarcted region at 1 day post‐MI.^40^ Likely both scenarios coexist, whereby epicardial cells in regions most affected undergo cell death, while spared cells rapidly expand and regenerate. Following extensive depletion of the ventricular epicardium in zebrafish, spared epicardial cells showed a remarkable ability to repopulate the layer.^75^ Hedgehog signaling promoted epicardial proliferation in this model.^75^ This pathway, among others, should be explored in the mammalian system to reveal new targets to enhance epicardial activation and extend the reparative window to adult mammals.

Along with epicardial reactivation, injury stimulates expansion of the subepicardial mesenchyme, analogous to that which forms and functions during embryonic heart development.^18^, ^65^ Yet, its roles in cardiac repair are incompletely understood. Subepicardial tissue thickening positively correlates with cardiac function, especially when exogenously enhanced, e.g., with Tβ4 (Figure 2).^58^, ^59^, ^67^, ^68^ The subepicardial space accommodates neovascularization, by vessel sprouting,^54^ expansion of preexisting endothelial cells,^53^ and assembly of collateral^76^ and lymphatic networks.^77^ In addition to revascularization, the newly synthesized matrix may temporarily stabilize the myocardial wall, akin to early reparative fibrosis^9^, ^78^ and, at least in regenerative models, provides matrix that favors CM repopulation^79^, ^80^, ^81^; however, this has yet to be confirmed for the mammalian epicardium. Indeed, the de novo epicardium‐derived tissue that is characteristically mesenchymal and often marks the epicardial response after cardiac injury^52^, ^53^, ^78^ is poorly researched in adult mice.^82^ Whether it is established through partial vs complete EMT or morphogenic vs fibrogenic EMT^83^ remains unclear. Recent guidelines aimed at assessing EMT and harmonizing definitions across the field^83^ remind us that most epicardial EMT studies fall short. The EMT process represents a spectrum with multiple E‐to‐M states—both in development and injury^83^—which also allows for collective epicardial cell migration by leader cells with quasi‐mesenchymal phenotype.^84^, ^85^ Difficulties in assessing whether epiEMT is complete or if epicardial cells—in vitro or in vivo*—*exist in one of several transitory states at any given time may contribute to perceived heterogeneity in epicardial gene expression and function.^85^, ^86^, ^87^, ^88^ The use of common epicardial markers similarly adds to this distraction, as they alone cannot distinguish epicardial cells from their transitory and differentiated progeny, as noted above. Further confounders are introduced in cell culture studies, where cell density, ECM composition and interaction with other cell types profoundly influence epicardial‐mesenchymal plasticity. In vitro conditions are shown to favor mesenchymal states, increasing phenotypic heterogeneity with culture.^89^ Any in vitro studies on epiEMT and ECM production require rigorous validation in vivo. Characterizing the nature of epiEMT is as important as identifying the factors stimulating the process. Comprehensively defining the cellular and ECM composition of the (sub)epicardial tissue will inform therapeutic strategies to maximize reparative potential.

**FIGURE 3:**
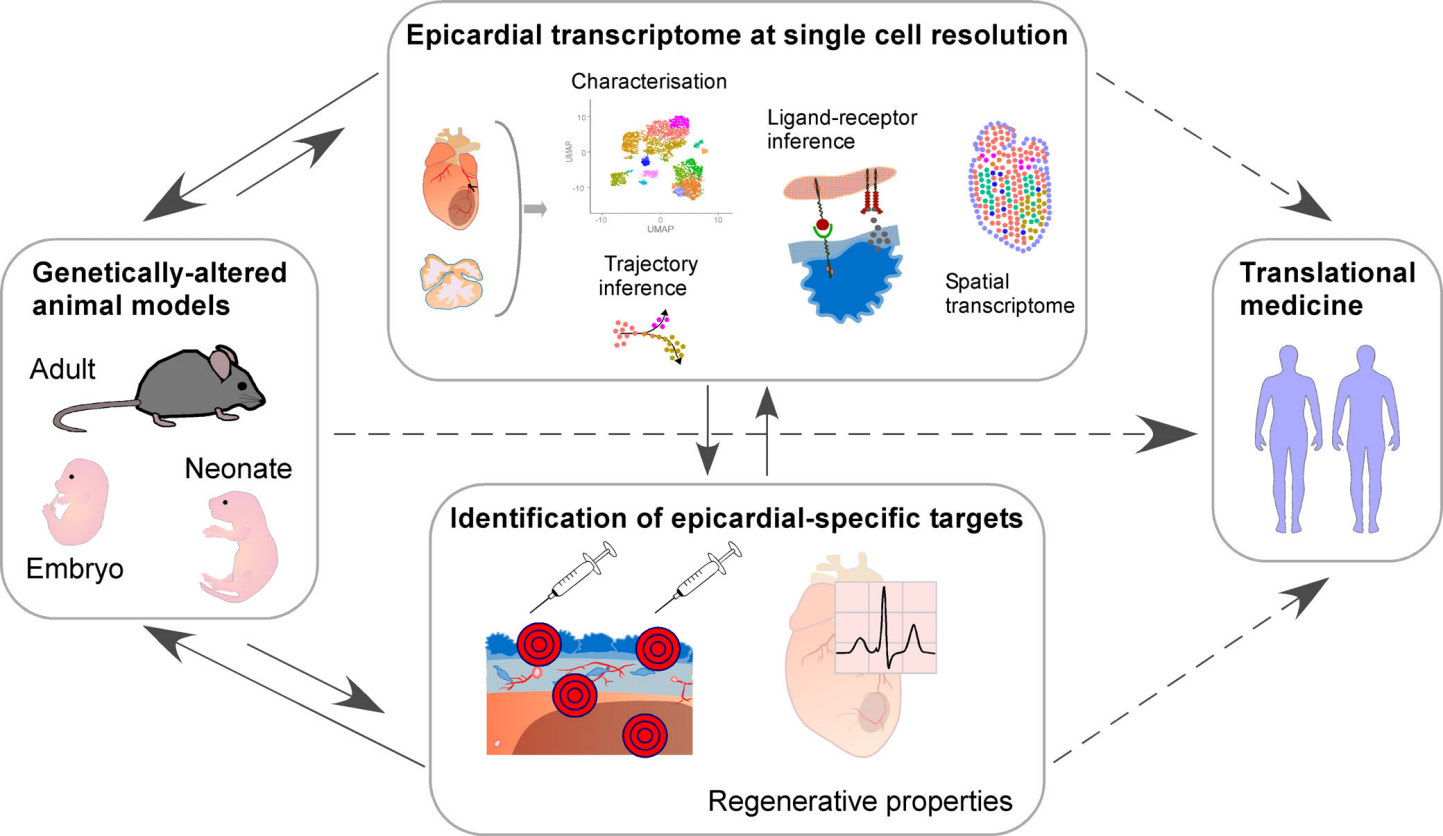
A workflow integrating single‐cell resolution transcriptomics to facilitate research into epicardial biology. Recent technological advances will enable characterization of the epicardial transcriptome, both in development and injury, from which we may infer lineage trajectories and signaling. Epicardial targets and markers validated in new genetically altered animal models will facilitate development of potential new treatments to restore regenerative capabilities

Increasingly, epicardial processes are being examined with “omic” analysis of in vivo samples, followed by requisite in situ validation and knockout studies. Only a handful have attempted to profile the epicardial transcriptome throughout development and injury,^50^, ^73^, ^86^ many of which were critically hampered by the combined inclusion of nonepicardial cells or epicardial cells of indiscriminate E‐to‐M states within samples. With more research groups employing improved scRNA‐seq platforms to profile cardiac cells,^47^ it is a matter of time before single‐cell resolution profiling of adult epicardial cells from healthy and diseased hearts is realized (Figure 3). Powerfully combined with lineage tracing, epicardial cells and their derivatives may be rigorously characterized for differential gene expression, E‐to‐M and extrapolated cell fate trajectories. Several epicardial studies have inferred embryonic stage‐specific functions using scRNA‐seq data.^18^, ^41^, ^90^ In contrast, less is known of neonatal and adult epicardium as, unsurprisingly, cardiac injury studies focused primarily on more abundant populations, such as CMs and CFbs.^8^, ^91^, ^92^, ^93^ At day 7 post‐MI, Farbehi et al^8^ detected a *Pdgfra*+ population with epicardial signature (*Wt1*+; ~1% of total *Pdgfra*+ cells), which expressed genes such as *Col1a1*, *Fn1*, and *Postn* ^8^ resembling the epicardium‐derived mesenchymal cells of the adult subepicardium. At day 14 post‐MI, DePasquale et al^94^ demonstrated putative regulatory interactions among differentially expressed genes in the epicardial cluster of MI vs sham hearts. Early growth response 1 (*Egr1*) formed a central transcriptional node in this gene network and appears to be implicated in downregulation of the mesothelial gene *Upk3b*,^94^ suggestive of E‐to‐M states post‐MI. Future scRNA‐seq studies may benefit from flow cytometry‐based methods to enrich for the epicardium and its derivatives, and use of sensitive and high read depth sequencing to rigorously analyze differential expression post‐MI. Spatial transcriptomics, previously utilized to study the developing human heart,^95^ may also be employed to interrogate remote and infarct zone epicardial cells in cardiac injury.

Taken together, the picture that emerges of the epicardium in endogenous repair of the injured mammalian heart consists of remodeling of subepicardial matrix and paracrine stimulation of new vessel growth, immunomodulation, and limited cellular contribution. While epicardial‐secreted growth factors reduced infarct size,^53^ this appears to be via enhanced CM survival and improved neovascularization, without a significant degree of CM replenishment, as occurs in zebrafish,^1^, ^16^ for example. Therefore, the therapeutic prospects of epicardial‐based repair hinges upon in situ reactivation and modulation of epicardial fate or on cell transplantation and tissue engineering approaches.

## 3 EVALUATING THE TRANSLATIONAL POTENTIAL OF THE EPICARDIUM

Therapeutic targeting of the epicardium for cardiac regeneration in animal models^58^, ^59^, ^66^, ^67^, ^96^, ^97^, ^98^ has generated much enthusiasm for its translation to human patients. An early candidate for boosting epicardial activity was Tβ4, with both pre‐MI^58^, ^67^ and post‐MI treatment^59^, ^66^ of mice leading to improved cardiac functional recovery. Whether or not the epicardium provides de novo specialized cells, and what type, varies according to the exogenous treatment.^52^, ^53^, ^58^, ^59^ Nevertheless, common between treatments is the enhancement of subepicardial thickness and apparent epicardial‐enhanced neovascularization (Figure 2).^54^, ^58^, ^59^, ^66^, ^67^, ^96^, ^98^ Epicardial VEGF, Tβ4, and PKR signaling have associated roles in embryonic coronary vasculogenesis.^99^, ^100^, ^101^ Continued research on the intrinsic adult epicardial response, and comparison with development, will undoubtedly reveal more targets, potentially for combinatorial treatment strategies. Once these are established, the next questions will relate to translation in humans and effective delivery methods.

Epicardial‐like cells have been generated in vitro from human pluripotent stem cells (hPSCs) by replicating successive, developmentally relevant transitions through growth factor‐controlled lineage specification: induction of LPM formation with BMP4, either with^102^ or without^103^ FGF2, and differentiation toward the epicardial lineage by a combined activation of WNT and BMP signaling.^102^, ^103^ hPSC derivatives can be further differentiated toward a vSMC or CFb fate using the growth factors identified from animal studies (vSMCs: various combinations of TGFβ1, PDGF‐BB, and FGF2; CFbs: FGF2 with or without VEGFA).^102^, ^103^ Human epicardial cultures, whether primary^104^ or stem cell derived, offer a platform for drug screening and proof of concept investigation of molecular mechanisms. With their human origin, it may be argued that they are translationally more relevant. However, more specific markers are required to distinguish epicardial cells from their derivatives, as mixed cell states coexist in culture.^87^, ^89^, ^105^ The phenotype of these cells will ultimately be dictated by culture conditions and the extent to which they resemble human epicardium in vivo is difficult to ascertain.

hPSC‐derived epicardial‐like cells have been powerfully applied to regenerative strategies, with their incorporation into engineered heart tissue in vitro and co‐transplantation with hPSC‐derived CMs into infarcted rat hearts.^105^ Co‐transplantation enhanced cardiac graft size and systolic function, compared with either cell type alone; however, the mechanism of the observed synergy is incompletely understood. Paracrine secretion of trophic factors from a “fetal‐like” epicardium correlated with enhanced CM survival and/or proliferation, neovascularization and synthesis of a modified ECM, particularly rich in fibronectin.^105^ Collectively, these properties would be expected to promote optimal repair; however, the relative contribution of each process remains to be determined. Moreover, the application of hSPC derivatives for cell therapy faces challenges, relating not only to phenotypic characterization, but to stemness, immaturity of derivatives and delivery/retention, as commonly encountered with other cell therapy candidates for cardiac regeneration.^106^ Consequently, a deeper understanding of the paracrine and ECM modulatory benefits of epicardial cell therapy may allow for reproducing the effects using a cocktail of paracrine factors, to obviate the difficulties of cell transplantation.

## 4 CONCLUSION

De novo contribution of epicardium‐derived cells took central stage in the early years of research into the injury‐activated adult epicardium. However, controversies surrounding epicardial fate, both in development and injury, highlight issues with current tools and urges the field to compromise or find alternatives. Recent studies have presented strong evidence to suggest that CM and CEC contribution is unlikely and, when therapeutic strategies are applied (principally Tβ4), CM differentiation is rare and dependent upon prophylactic “priming” of the epicardium,^58^, ^59^ which may limit therapeutic application. Only with better understanding of what the epicardium can achieve might we tailor treatment and manipulate the epicardium to augment myocardial survival and neovascularization. With the future in mind, we will need to ensure that progress into epicardial‐mediated regeneration is not hindered by poorly defining epicardial cells and use of outdated or unsuitable animal models. Furthermore, we should also avoid overly generalized assumptions when applying embryonic epicardial biology without establishing similarities and differences in the adult setting. Intrinsic adult epicardial activity will consist of distinct biological processes, rather than a complete recapitulation of the embryonic program. A consensus exists in support of the reparative properties of the epicardial secretome, and future research focusing on this trait will identify new and improved targets. Moreover, recent studies have uncovered an important, but barely understood, interaction between the epicardium and the inflammatory response after injury,^40^, ^64^ which appears to strongly influence the outcome in terms of repair and functional recovery. Due to the diverse array of beneficial effects it promotes, the epicardium remains an attractive target for cardiac regeneration. However, a greater understanding is required of the endogenous repair mechanisms and of stimuli that can modulate these processes for enhanced repair. Ultimately, it may be possible to differentially control individual components of the epicardial repertoire, which may sufficiently promote repair, or may be targeted alongside therapies that promote CM proliferation and immunomodulation.

## CONFLICT OF INTEREST

The authors declared no potential conflicts of interest.

## AUTHOR CONTRIBUTIONS

A.N.R: writing‐original draft and figure preparation; A.N.R., N.S.: writing‐review and editing, final approval of the manuscript.

5

## Data Availability

Data sharing is not applicable to this article as no new data were created or analyzed in this study.
